# Colorimetric determination of carbidopa in anti-Parkinson drugs based on 4-hydroxy-3-methoxybenzaldazine formation by reaction with vanillin

**DOI:** 10.1007/s00216-022-04256-4

**Published:** 2022-08-04

**Authors:** Mariagrazia Lettieri, Simona Scarano, Pasquale Palladino, Maria Minunni

**Affiliations:** Department of Chemistry ‘Ugo Schiff’, University of Florence, Via della Lastruccia 3-13, 50019 Sesto Fiorentino, Italy

**Keywords:** Anti-Parkinson drugs, Vanillin, Benzaldazine, Levodopa, Carbidopa, Colorimetry

## Abstract

In this paper is reported the selective colorimetric detection and quantification of carbidopa, an inhibitor of aromatic amino acid decarboxylase, in the co-presence of levodopa as dopamine precursor in pharmaceutical formulations for the treatment of Parkinson’s disease. The method is based on the selective condensation reaction between the hydrazine group from carbidopa and the formyl functional group of vanillin, a natural flavoring agent, in acidified alcoholic solution. The yellow color development (*λ*_max_ ~ 420 nm) due to the formation of 4-hydroxy-3-methoxybenzaldazine (HMOB) was observed for carbidopa only, whereas levodopa, lacking the hydrazine group, did not color the solution, as expected. The calibration curves for two tablet formulations of levodopa in combination with carbidopa (4:1) were superimposable with levodopa/carbidopa (4:1), as well as carbidopa alone, in standard solution, i.e., the excipients and additives did not interfere with carbidopa determination, corresponding to a mean recovery about 105%. The linear dynamic range was between 5.00 and 50.0 mg L^−1^ with very good reproducibility within this range (CV_av_% about 3–4%) and very good sensitivity, with limits of quantification of about 1 mg L^−1^. The colorimetric method developed here is very simple, inexpensive, and effective for drug estimation and quality control of pharmaceutical formulations.

**Graphical abstract:**

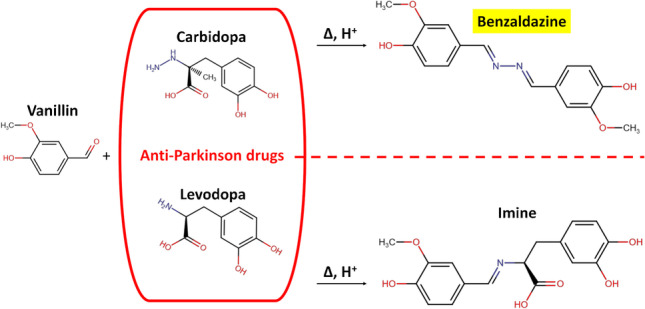

**Supplementary Information:**

The online version contains supplementary material available at 10.1007/s00216-022-04256-4.

## Introduction

**Fig. 1:**
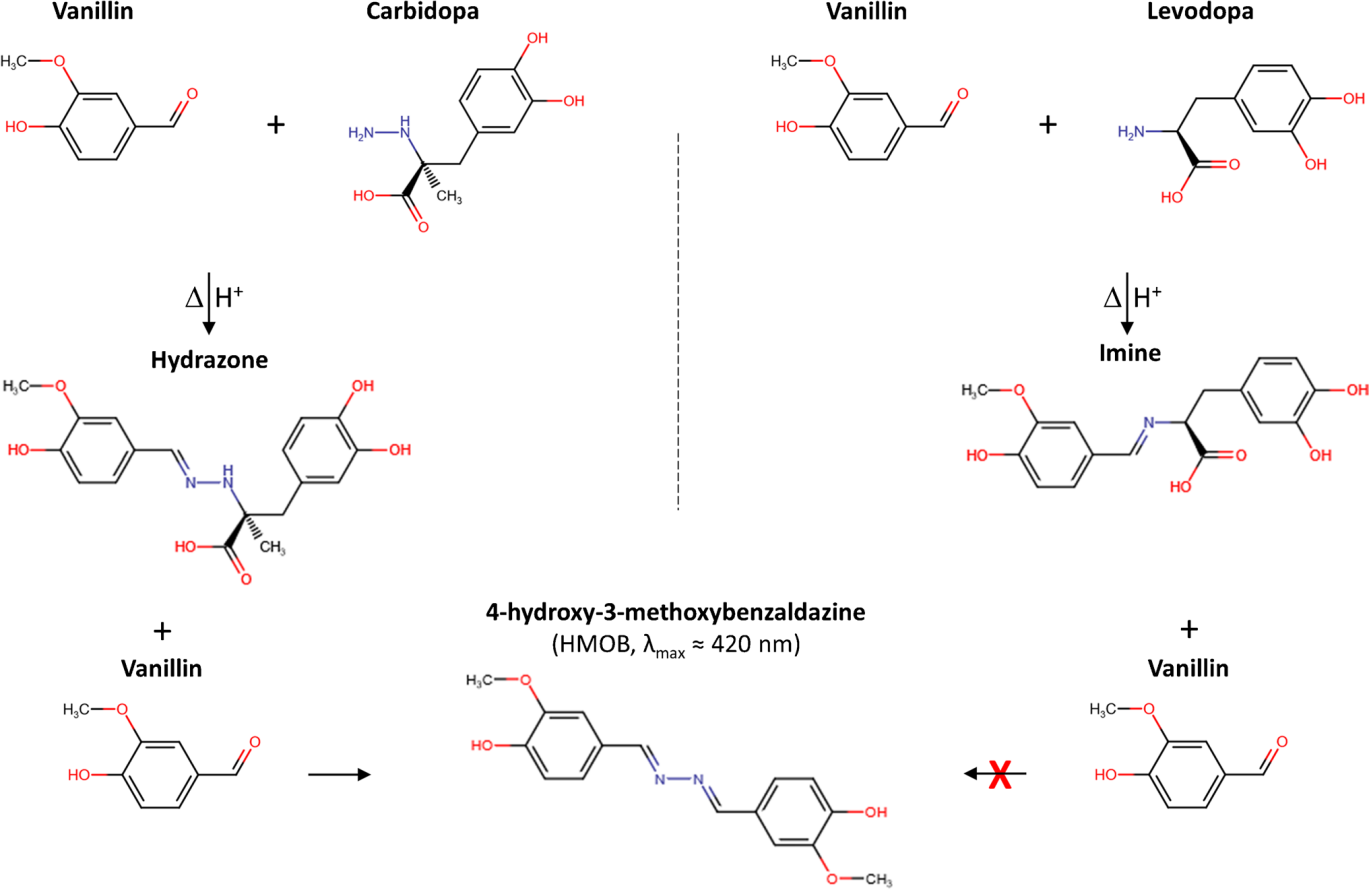
Scheme of condensation reactions of vanillin with carbidopa and levodopa. Carbidopa hydrazide functional group produces a hydrazone that further reacts with an excess of vanillin to form the 4-hydroxy-3-methoxybenzaldazine (HMOB), with a maximum visible absorbance about 420 nm (yellow). Levodopa amine group produces an imine that cannot generate the azine, leaving the solution uncolored at acidic pH

The Parkinson’s disease (PD) is associated with abnormal dopaminergic neuron disruption and neurotransmitters loss and conversion into cytotoxic molecules, affecting 10 million people worldwide [1–4]. At the present time, there is no cure for PD, but the administration of some medications to restore the dopamine (DA) concentration in the brain may improve, although variably and transiently, the quality of life of patients [5, 6]. Unfortunately, DA cannot be directly taken as a medicine because it is unable to reach the brain. Accordingly, the pharmacological treatment of PD is based on levodopa (LD), which is the molecular precursor of DA, together with carbidopa (CD), which works as peripheral inhibitor of aromatic amino acid decarboxylase, avoiding the conversion of LD in DA prior to crossing the blood–brain barrier, thus largely reducing the dose, and the resulting side effects, of levodopa to be administered. Several methods were developed for CD determination in presence of LD in such drugs by means of chromatography [7], electrochemistry and electrophoresis [8, 9], spectrophotometry [10–12], and NMR [13]. Recently, we have reported the selective detection and quantification of levodopa in co-presence of carbidopa in these commercial drugs for the treatment of PD. The method was based on the generation and stabilization of the purple melanochrome (*λ*_max_ ~ 585 nm) from levodopa by using magnesium acetate and dimethyl sulfoxide [14]. Here, instead, we report the detection and quantification of carbidopa for the same tablet formulations, exploiting the well-known and selective reactivity of hydrazine group of carbidopa with an (aromatic) aldehyde. At the same time, the levodopa, lacking the hydrazine group, forms only an imine and leaves the solution not colored at acidic pH. We decided to use vanillin (4-hydroxy-3-methoxybenzaldehyde), observing the yellow color development (*λ*_max_ ~ 420 nm) due to formation of 4-hydroxy-3-methoxybenzaldazine by reaction with carbidopa (Fig. 1). The same azine was previously obtained by the condensation of free hydrazine molecule with vanillin: (a) as a probe to develop a colorimetric hydrazine dosimeter [15]; (b) as a Schiff base ligand to generate biologically active transition metal complexes [16]; (c) as side-products [17]. Moreover, an analogous azine was obtained by reaction of carbidopa with p-dimethylaminobenzaldehyde (DMAB) for spectrofluorometric determination of carbidopa in monkey plasma [18]. However, being vanillin a well-known water-soluble and naturally occurring flavoring agent used in food industries [19, 20], although adverse effect can occur due to chemical reactivity, it appears a safer and convenient alternative to the synthetic and low water-soluble DMAB. Based on this, we decided to develop a colorimetric quantitative assay for CD estimation, offering a simple, fast, and low-cost alternative to the other methodologies requiring large instrumentation, for control of pharmaceutical formulations also in advanced drug delivery systems [21]. We explored the chemical determinants of color development for CD by changing vanillin concentration, solvent composition and pH, heating temperature and time, as detailed in the following sections, focusing the study on the detection of CD in combination with LD as found in some pharmaceutical formulations (Sinemet and Hexal) for the treatment of parkinsonism. Major efforts were devoted to obtaining large, selective, and reproducible color development from CD solutions, avoiding at the same time both the possible interference from the pharmaceutical additives and the spontaneous oxidation and polymerization of LD, typical of catecholamines, which gives rise to a black/brown polymer with a broad absorbance spectrum, including the visible region, therefore potentially affecting the colorimetric assay [22–26]. We found the generation of the yellow color from carbidopa and uncolored solutions for levodopa in the same buffer conditions, irrespective of the notable redox properties of LD [14]. Therefore, we focused our investigations on the selective detection and quantification of CD in some anti-Parkinson tablet formulations at a mass ratio of 4:1 (LD 200 mg + CD 50 mg), together with numerous excipients. The modulation of yellow color intensity upon vanillin reaction was explored up to 200 mg L^−1^ LD and 50.0 mg L^−1^ CD. The calibration curves for two tablet formulations were superimposable to levodopa/carbidopa (4:1), as well as to CD alone, showing a linear dynamic range between 50.0 and 5.00 mg L^−1^ of CD with very good reproducibility within this range and a good mean recovery of CD for both drugs. Data were acquired by using a common microplate reader, underlining the affordability of the method for drug estimation and quality control of pharmaceutical formulations. Furthermore, based on the very good sensitivity, for brand and generic drugs, this assay could pave the way for future research into therapeutic monitoring area for carbidopa-based medications.

## Materials and methods

### Chemicals

Carbidopa (CD, (2S)-3-(3,4-dihydroxyphenyl)-2-hydrazinyl-2-methylpropanoic acid), levodopa (LD, (2S)-2-amino-3-(3,4-dihydroxyphenyl)propanoic acid), vanillin (4-hydroxy-3-methoxybenzaldehyde), acetonitrile (MeCN), ethanol (EtOH), dimethyl sulfoxide (DMSO, methylsulfinylmethane), methanol (MeOH), and hydrogen chloride (HCl) were purchased from Sigma-Aldrich (Milan, Italy). Brand drug Sinemet (200 mg levodopa, 50 mg carbidopa, 35 mg excipients) was obtained from MSD (Rome, Italy), and its pharmaceutical alternative (generic drug) Hexal (200 mg levodopa, 50 mg carbidopa, 194 mg excipients) was produced by Sandoz (Basel, Switzerland). All chemicals were of analytical reagent grade and used as received without any further purification. All solutions were prepared using water obtained from Milli-Q Water Purification System (resistivity ≥ 18 MΩcm) (Germany, www.merckmillipore.com).

### Methods and instrumentation

Carbidopa and levodopa were dissolved in HCl 1.00 M to obtain 100 g L^−1^ stock solutions. Stock solutions of a brand drug and the generic drug containing 5.00 g L^−1^ of levodopa were prepared by stirring for 10 min one tablet (200 mg LD, 50 mg CD) in 40.0 mL of 1.00 M HCl. The working solutions were prepared within 24 h from tablet dissolution. The samples were heated by using thermomixer to control time and temperature of the reaction. All the solutions in different solvents (acetonitrile, methanol, ethanol, water, dimethyl sulfoxide, and EtOH:H_2_O mixtures) contained 50.0 mg L^−1^ (0.221 mM) of CD and 10.0 mM vanillin. The same CD and vanillin concentrations were used for EtOH:H_2_O (1:1) solutions containing HCl 5 mM, 50 mM, or 500 mM. In all these cases the absorbance spectra were acquired after 4 h at 70 °C. The condition of CD 50.0 mg L^−1^ in EtOH/H_2_O 1:1 with HCl 0.500 M at 70 °C was applied also to different vanillin concentrations (0.00, 1.25, 2.50, 5.00, 10.0, 20.0 mM) for 4 h: vanillin 10.0 mM at 30 °C, 40 °C, 50 °C, 60 °C, 70 °C, 80 °C for 4 h; vanillin 10.0 mM at 70 °C for 15, 30, 60, 120, 240 min. For calibration curves, each concentration of CD, LD + CD 4:1, or pharmaceutical formulations was in HCl 1.00 M, and added then to vanillin 20.0 mM in ethanol, obtaining the final sample solutions in EtOH:H_2_O 1:1, HCl 0.500 mM and vanillin 10.0 mM. The limit of detection (LOD) and the limit of quantification (LOQ) were calculated based on the standard deviation (SD) of the mean of the blank values, as 3 × SD/*m* and 10 × SD/*m*, respectively, where *m* indicates the slope of the calibration curve. The assay reproducibility is reported as (mean) coefficient of variation (CV_av_). Absorbance spectra were acquired in disposable polystyrene 96-well microtest plates (Sarstedt, Milan, Italy) by using iMark™ microplate visible absorbance reader with optical filters (Bio- Rad, Milan, Italy), as well as in 1.0 cm cell by using a UV–Visible Spectrophotometer Evolution™ 201/220 from Thermo Scientific™ (Rodano, Milan, Italy).

### Assay principle

Pioneering works described the reaction between hydrazine and an acidified alcoholic solution of p-dimethylaminobenzaldehyde (DMAB), resulting in the development of a characteristic yellow color that was used for the quantitative determination of hydrazine and hydrazide derivatives [18, 27–29]. More in general, the reactivity of the hydrazine, or hydrazide group, allows the formation of hydrazone by 1:1 by condensation with an aldehyde [15, 30, 31]. The hydrazone can further react with an aldehyde to form an azine [16–18, 32, 33] with several applications, ranging from material chemistry to medicinal chemistry [33]. When the aldehyde is aromatic, like a benzaldehyde derivative, the product of condensation resulted colored and fluorescent, depending on several factors, including the ring substitutions and solvent polarity [18, 32]. Levodopa, instead, can give an aldimine (Schiff base) by amine/aldehyde condensation reaction, but cannot lead to azine formation, lacking the hydrazine group and therefore leaving the solution not colored at acidic pH, whereas at basic pH, LD, as other catecholamines (dopamine, norepinephrine, methyldopa), can form yellow unstable aminochrome derivatives [3, 14, 34, 35]. Grounded on this, we decided to develop a colorimetric assay for carbidopa quantification based on the reaction of an excess of vanillin (4-hydroxy-3-methoxybenzaldehyde) with the hydrazide group of carbidopa at acidic pH to generate the yellow 4-hydroxy-3-methoxybenzaldazine (HMOB, *λ*_max_ ~ 420 nm in ethanol/water solution) and 3,4-dihydroxyphenylacetone in Fig. 1 [36, 37].

## Results and discussion

### Influence of solvent composition and pH on color development

**Fig. 2:**
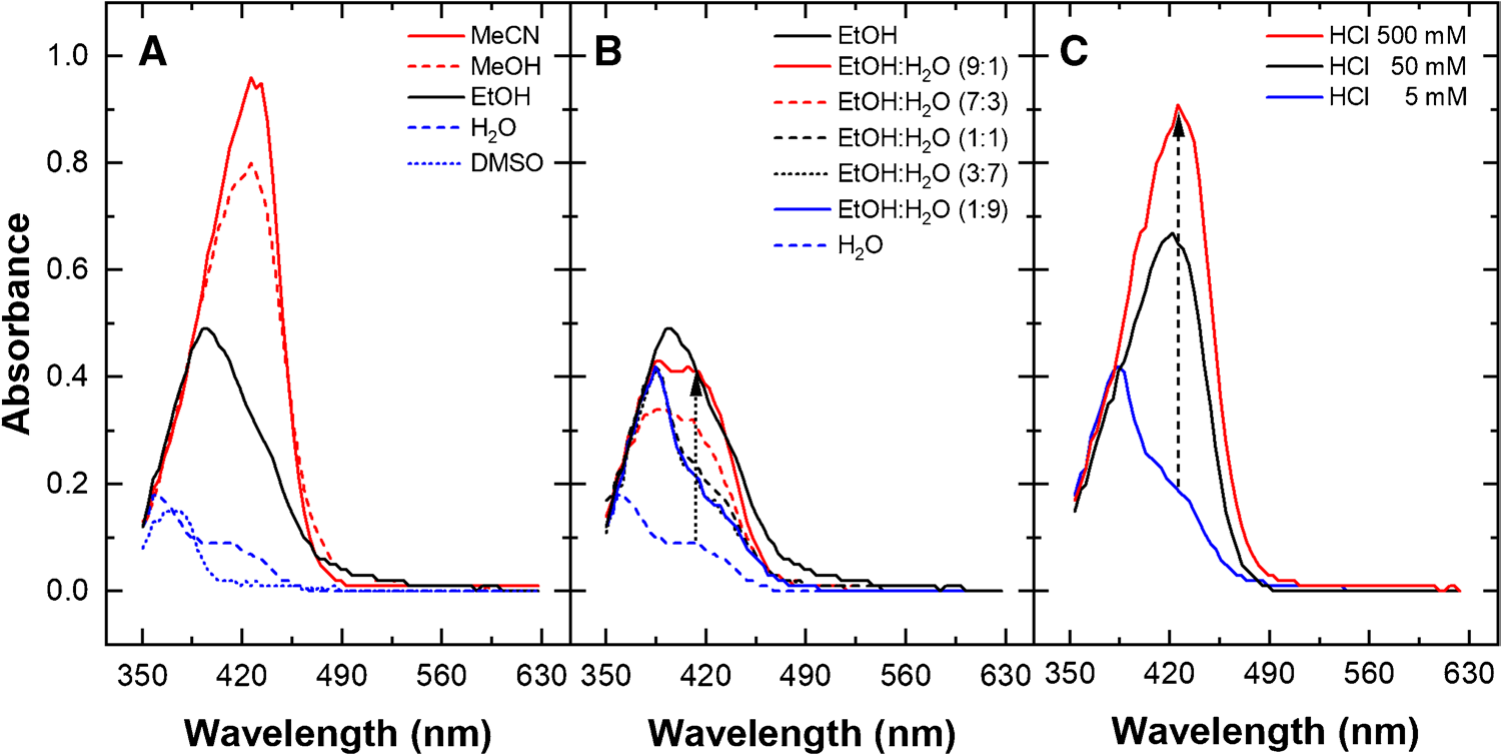
Influence of solvent composition and pH on color development. All the solutions contain 50.0 mg L^−1^ (0.221 mM) of CD and 10.0 mM vanillin reacted for 4 h at 70 °C. (**A**) Absorbance spectra in acetonitrile (MeCN, red line), methanol (MeOH, red dashes), ethanol (EtOH, black line), water (H_2_O, blue dashes), dimethyl sulfoxide (DMSO, blue dots). (**B**) Absorbance spectra for different EtOH:H_2_O compositions, from neat water (H_2_O, blue dashes) to neat ethanol (EtOH, black line). (**C**) Absorbance spectra in EtOH:H_2_O (1:1), containing HCl 5 mM (blue line), 50 mM (black line), or 500 mM (red line)

Several experimental parameters of HMOB formation were tested here by using a spectrophotometer or a microplate reader for visible absorbance detection. Firstly, the role of solvent composition and pH on carbidopa/vanillin reaction at 70 °C for 4 h was evaluated for 50.0 mg L^−1^ (0.221 mM) of CD in presence of a large excess of vanillin (10.0 mM) by using absorbance spectra acquired in quartz cuvettes. Figure 2A shows that the absorbance of the solution decreased with the polarity of the solvent, reaching the largest values for acetonitrile or methanol, and the lowest values for H_2_O or DMSO. Pure ethanol appeared as a good compromise between sensitivity and health risk. Accordingly, we decided to continue the method development based on ethanol:water solutions. Figure 2B reports the results of the same reaction for different compositions of EtOH:H_2_O as solvent system. The color development was proportional to ethanol content, as expected from Fig. 2B. Interestingly, absorbance spectra for EtOH:H_2_O 3:7 and 1:1 appeared almost superimposable, and this aspect resulted very useful to develop a robust assay with a minimal dependence on possible variation of solvent composition. Therefore, we focused on EtOH:H_2_O (1:1) solvent system, exploring the dependence of the reaction on the acidity of the solution. Figure 2C shows the large intensity increase obtained incrementing the HCl content from 5 to 500 mM, with a *λ*_max_ about 420 nm.

### Influence of vanillin concentration, temperature, and heating time on color development

**Fig. 3:**
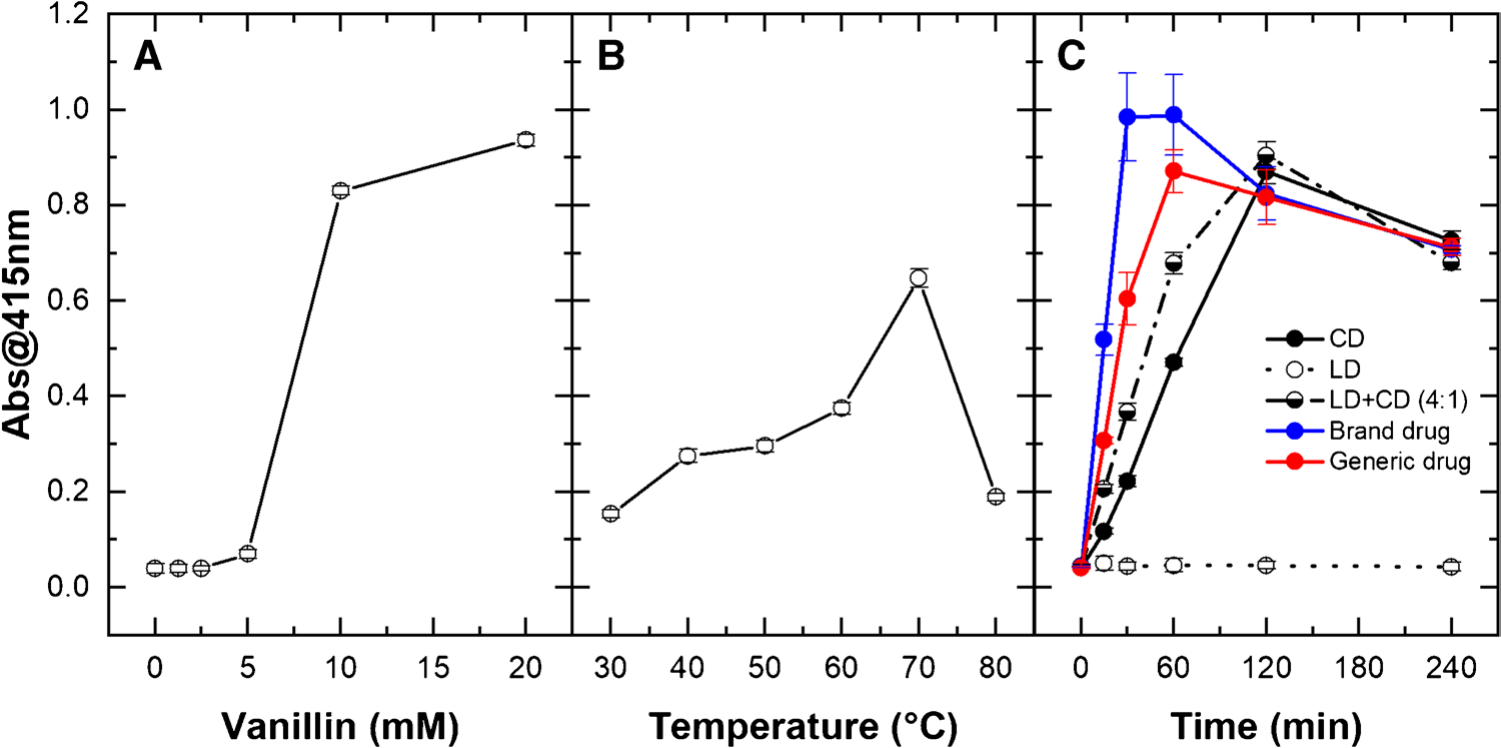
Influence of vanillin concentration, temperature, and heating time on color development. Absorbance at 415 nm for CD 50.0 mg L^−1^ samples dissolved in EtOH/H_2_O 1:1 with HCl 0.500 M. (**A**) Vanillin 0.00, 1.25, 2.50, 5.00, 10.0, 20.0 mM after 4 h at 70 °C. (**B**) Vanillin 10.0 mM at 20 °C, 30 °C, 40 °C, 50 °C, 60 °C, 70 °C after 4 h. (**C**) Vanillin 10.0 mM at 70 °C after 15, 30, 60, 120, 240 min in presence of carbidopa (CD, black circles), levodopa (LD, white circles), LD + CD 4:1 (half black circles), brand drug (blue circles), and generic drug (red circles). Each point represents the mean ± SD of 4 replicates

Subsequent measurements were performed by using iMark™ Microplate Absorbance Reader determining the absorbance values for 0.200 mL solutions in disposable polystyrene 96-well microtest plates at 415 nm, i.e., the available optical filter closest to the absorbance *λ*_max_ about 420 nm. Figure 3 shows the results of the experiments for CD 50.0 mg L^−1^ dissolved in EtOH /H_2_O 1:1 with HCl 0.500 M. Figure 3A shows the effect of vanillin concentration (0.00, 0.50, 2.5, 4.4, 10.0, 20.0 mM) on color development after 4 h at 70 °C. The color change was almost undetectable by vanillin addition up to 5.00 mM; a large absorbance increase occurred for vanillin between 5.00 and 10.0 mM, and further increase for vanillin at 20 mM. Considering that carbidopa is fixed at 50.0 mg L^−1^ (0.221 mM), this results underlining the need of a large excess of vanillin to obtain the condensation reaction described in Fig. 1, which is responsible for HMOB formation and color development. Vanillin at 10 mM appears as the best concentration in terms of data reproducibility (CV% 1.1). Figure 3B shows that the absorbance recorded at 415 nm increases with the temperature, reaching the maximum at 70 °C, and then falling at 80 °C. Although the reproducibility is very good at any temperature, 70 °C appeared the best choice (CV% 2.9). Finally, Fig. 3C reports the absorbance of the CD solutions that increased with the heating time up to 2 h, and then decreased up to 4 h in these conditions. Data reproducibility was very good for carbidopa at any reaction time. However, the advantage to use 4 h for color development was clear when we applied the same procedure to levodopa/carbidopa as mixed standards or in real drugs (Fig. 3C). In detail, apart from LD alone that gave no color development, as expected, all mixtures containing CD gave rise to yellow solutions but with a much worse reproducibility and faster kinetics with respect to carbidopa alone. Such discrepancy among the absorbance values decreased at 2 h, reaching a minimum after 4 h, as evident from the data superposition in Fig. 3C for CD, LD + CD, brand drug, and generic drug, indicating this reaction time as the best option to build a calibration curve to estimate the carbidopa concentration in pharmaceutical formulation, as detailed below.

### Colorimetric quantification of carbidopa in tablets by using of HMOB as molecular probe

**Fig. 4:**
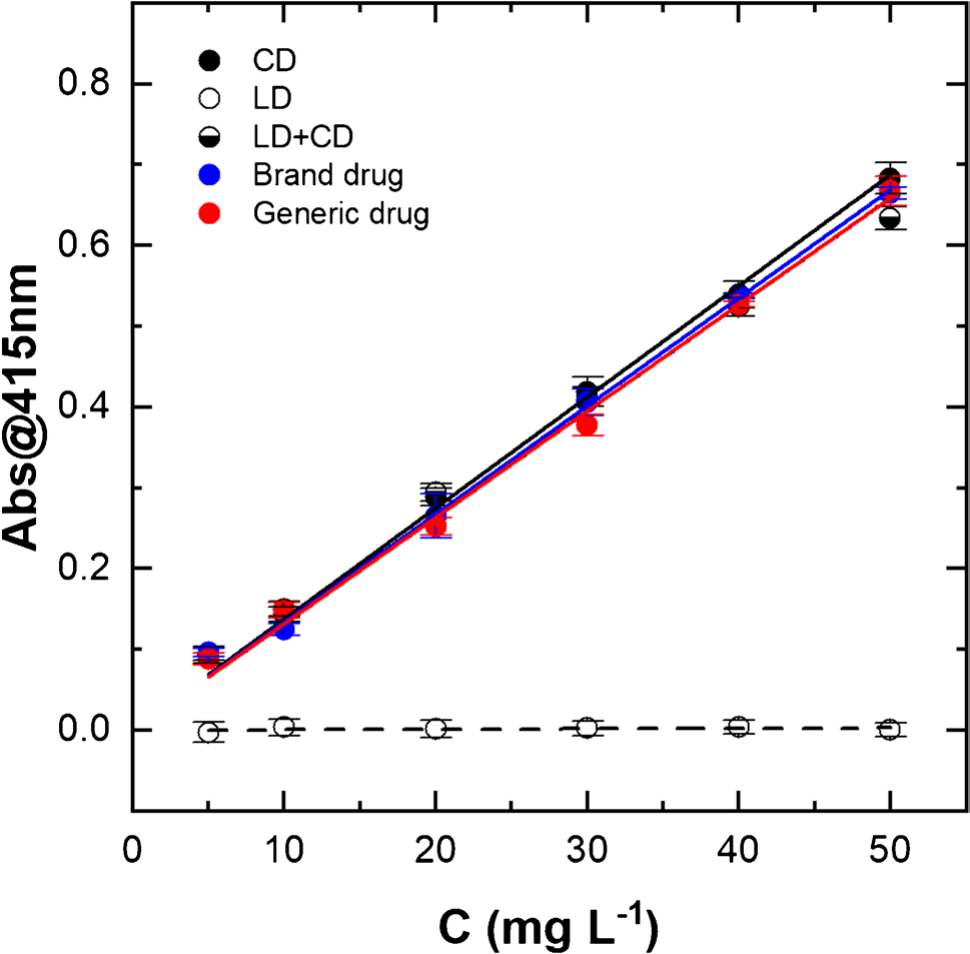
Colorimetric calibration plots. Absorbance values at 415 nm for sample solutions in EtOH/H_2_O 1:1 with HCl 0.500 M and vanillin 10.0 mM after 4 h. Carbidopa (CD, black circles), levodopa (LD, white circles), LD + CD 4:1 (half black circles), brand drug (blue circles), and generic drug (red circles). The explored concentration range was between 5.00 and 50.0 mg L^−1^ for CD, and between 20.0 and 200 mg L^−1^ for LD alone or in combination with CD. Each point represents the mean ± SD of 4 replicates. Data were fitted according to linear Eq. 1

The best experimental parameters for HMOB formation and the concomitant color development from CD solutions in terms of sensitivity and reproducibility were identified here as 10.0 mM vanillin in presence of 0.500 M HCl in EtOH/H_2_O 1:1 (v/v) at 70 °C for 4 h. Accordingly, these parameters were fixed to selectively detect CD in co-presence of LD, as for the pharmaceutical formulations for the treatment of parkinsonism. Figure 4 reports the calibration curves for CD (black circle), LD (white circles), the binary mixture of pure components (LD + CD 4:1) (black and white circles), brand drug (blue circles), and the generic drug (red circles). In detail, LD standard solutions did not show any absorption at 415 nm over the entire concentration range here explored, as expected due to the absence of the hydrazine group, preventing the synthesis of the HMOB (Fig. 1). Differently, the LD + CD 4:1 mixture, which represents a simplified model of commercial drugs, and the levodopa/carbidopa tablets, which represent the real samples, containing 200 mg of levodopa and 50 mg of carbidopa, together with the excipients (see “Materials and methods”), all gave colored solutions, as for CD standard solutions. After blank subtraction, data corresponding to CD between and 50.0 mg L^−1^ were fitted according to the linear equation

1 $$\mathrm{Abs}@415\mathrm{nm }= m \times C,$$

where *m* indicates the slope of the HMOB absorbance at 415 nm. The excellent performances of the assay are well highlighted by the superposition of the curves, due to the almost identical slope values reported in Table 1. Moreover, the analytical parameters from data fitting underline the absence of the matrix effect, due to the different formulations of such drugs, as well as the absence of interference from LD in such conditions, leading to a very good assay reproducibility and promising sensitivity (CV_av_% 3.7 and 3.4; LOD 0.215 ± 0.003 mg L^−1^ and 0.342 ± 0.006 mg L^−1^ for carbidopa in brand and generic drugs, respectively). Finally, Table S1 (see Electronic Supplementary Material) reports the recovery estimated by using the absorbance values for carbidopa alone with respect to pharmaceutical formulations at the corresponding nominal concentration of CD, obtaining a mean recovery value of 104.6% and 105.3% for brand and generic drugs, respectively, over the entire range of calibration.

**Table 1:** Linear fitting parameters for carbidopa quantification at 415 nm (Fig. 4)

Sample	^1^ m (L g^−1^)	^2^LOD (mg L^−1^)	^3^LOQ (mg L^−1^)	***R***^2^	^4^CV_av_ (%)
CD	13.7 ± 0.2	0.399 ± 0.005	1.330 ± 0.018	0.996	4.2
LD + CD	13.2 ± 0.3	0.184 ± 0.005	0.615 ± 0.015	0.987	3.5
Brand drug	13.4 ± 0.2	0.215 ± 0.003	0.778 ± 0.010	0.996	3.7
Generic drug	13.2 ± 0.2	0.342 ± 0.006	1.140 ± 0.019	0.995	3.4

^1^* m*, slope of the calibration curve. ^2^*LOD*, limit of detection. ^3^*LOQ*, limit of quantification. ^4^*CV*_*av*_, mean coefficient of variation calculated between 0.005 and 0.050 g L^−1^

## Conclusion

Carbidopa was added to levodopa and commercialized under the name of Sinemet since 1975, which represents the gold standard for the care of parkinsonism and generating, together with several other levodopa/carbidopa formulations, millions of prescriptions per year [4, 38]. Here, we reported the development of a colorimetric assay for the detection and quantification of carbidopa in such medications. The method is based on selective condensation reaction between carbidopa and vanillin, observing the yellow color development due to formation of HMOB, irrespective of levodopa content. The modulation of yellow color intensity upon vanillin reaction was explored up to 200.0 mg L^−1^ LD and 50.0 mg L^−1^ CD. The calibration curves for two tablet formulations were superimposable to levodopa/carbidopa (4:1), as well as to CD alone, showing a linear dynamic range between 50.0 mg L^−1^ and 5.00 mg L^−1^ of CD with very good reproducibility within this range (CV_av_%, 3–4%) and good mean recovery (105%). Data were acquired by using a common microplate reader, underlining the affordability of the method, appearing as an effective tool for drug estimation and quality control of pharmaceutical formulations. Moreover, considering that about 30% of carbidopa excreted in urine from patients that received an oral dose of such substance is not metabolized [36], the very good sensitivity of the assay, with limit of quantification about 1 mg L^−1^ for commercial drugs, stimulates a perspective study on non-invasive therapeutic monitoring of carbidopa-based medications in human excretion.

## Supplementary Information

Below is the link to the electronic supplementary material.

Supplementary file1 (DOCX 20 kb)
