# Supplementary material for: Colorimetric determination of carbidopa in anti-Parkinson drugs based on 4-hydroxy-3-methoxybenzaldazine formation by reaction with vanillin
Source: Anal Bioanal Chem. 2022 Aug 4;414(23):6911–8. doi: 10.1007/s00216-022-04256-4 (PMC9436860; doi:10.1007/s00216-022-04256-4)
Supplement: Supplementary file 1 — Supplementary file1 (DOCX 20 kb) [file 216_2022_4256_MOESM1_ESM.docx]

**Electronic Supplementary Material**

**Colorimetric determination of carbidopa in anti-Parkinson drugs**

**based on 4-hydroxy-3-methoxybenzaldazine formation by reaction with vanillin**

Mariagrazia Lettieri, Simona Scarano, Pasquale Palladino*, Maria Minunni

Department of Chemistry ‘Ugo Schiff’, University of Florence, Via della Lastruccia 3-13, 50019 Sesto Fiorentino, Italy

| **Table S1.** Absorbance values at 415 nm (Fig. 4) and corresponding CD recovery in drugs. | | | | | |
| --- | --- | --- | --- | --- | --- |
| **CD**  **(mg L^-1^)** | **Abs@415nm**  **CD** | **Abs@415nm**  **Brand drug** | **Recovery**  **(%)** | **Abs@415nm**  **Generic drug** | **Recovery**  **(%)** |
| 5.0 | 0.093 ± 0.010 | 0.096 ± 0.005 | 96.4 | 0.088 ± 0.007 | 105.7 |
| 10.0 | 0.143 ± 0.009 | 0.124 ± 0.007 | 115.3 | 0.149 ± 0.010 | 96.0 |
| 20.0 | 0.289 ± 0.011 | 0.265 ± 0.027 | 109.1 | 0.252 ± 0.011 | 114.7 |
| 30.0 | 0.419 ± 0.018 | 0.406 ± 0.016 | 103.2 | 0.378 ± 0.013 | 110.8 |
| 40.0 | 0.539 ± 0.016 | 0.538 ± 0.004 | 100.2 | 0.527 ± 0.003 | 102.3 |
| 50.0 | 0.683 ± 0.020 | 0.664 ± 0.008 | 102.9 | 0.668 ± 0.018 | 102.2 |
| Mean (%) |  |  | 104.6 |  | 105.3 |
